# Glucosinolates and Cytotoxic Activity of Collard Volatiles Obtained Using Microwave-Assisted Extraction

**DOI:** 10.3390/molecules28041657

**Published:** 2023-02-09

**Authors:** Azra Đulović, Franko Burčul, Vedrana Čikeš Čulić, Patrick Rollin, Ivica Blažević

**Affiliations:** 1 Department of Organic Chemistry, Faculty of Chemistry and Technology, University of Split, Ruđera Boškovića 35, 21000 Split, Croatia; 2 Department of Analytical Chemistry, Faculty of Chemistry and Technology, University of Split, Ruđera Boškovića 35, 21000 Split, Croatia; 3 School of Medicine, University of Split, Šoltanska 2, 21000 Split, Croatia; 4 Institute of Organic and Analytical Chemistry (ICOA), University of Orléans and the French National Center for Scientific Research (CNRS), UMR 7311, BP 6759, F-45067 Orléans, France

**Keywords:** collard, *Brassica oleracea* L. convar. *acephala* var. *viridis*, glucosinolates, isothiocyanates, microwave-assisted isolation, GC-MS, UHPLC-DAD-MS/MS

## Abstract

Glucosinolates (GSLs) in *Brassica oleracea* L. convar. *acephala* var. *viridis* (collard) flower, leaf, stem, and root were analyzed qualitatively and quantitatively via their desulfo-counterparts using UHPLC-DAD-MS/MS. Twelve GSLs were identified, including Met-derived GSLs (sinigrin, glucoibervirin, glucoerucin, glucoiberin, glucoraphanin, progoitrin), Trp-derived GSLs (4-hydroxyglucobrassicin, glucobrassicin, 4-methoxyglucobrassicin, and neoglucobrassicin), and Phe-derived GSLs (glucotropaeolin and gluconasturtiin). Total GSL content was highest in the root, having 63.40 μmol/g dried weight (DW), with gluconasturtiin (34.02 μmol/g DW) as the major GSL, followed by sinigrin and glucoibervirin (12.43 and 7.65 μmol/g DW, respectively). Total GSL contents in the flower, leaf, and stem were lower than in root, having 6.27, 2.64, and 1.84 μmol/g DW, respectively, with Trp and/or Met-derived GSLs as the predominant ones. GSL breakdown products were obtained via microwave hydrodiffusion and gravity (MHG) and volatile breakdown products were analyzed using GC-MS techniques. Volatile isolates were tested for their cytotoxic activity using MTT assay. MHG volatile extract from the root demonstrated the best cytotoxic activity against human bladder cancer cell line T24 and breast cancer cell line MDA-MB-231 during an incubation time of 72 h (IC_50_ 21.58, and 11.62 μg/mL, respectively). The activity of the root extract can be attributed to its major volatile, 2-phenylethyl isothiocyanate (gluconasturtiin breakdown product).

## 1. Introduction

*Brassica oleracea* is a vegetable crop with remarkable morphological diversity that comprises a number of popular vegetable cultivars such as kale, Brussels sprouts, broccoli, cauliflower, cabbage, collard greens, Savoy cabbage, kohlrabi, and gai lan. The inclusion of cruciferous vegetables in the diet of humans is reputed to be healthy, according to longstanding scientific research. Due to its beneficial effects on health, broccoli has been considered “the most popular” cruciferous vegetable. Its phytochemistry and biological activity have been well investigated and evaluated [1]. However, in recent years, the *acephala* group of these plants, which include the leafy, headless cabbages known as kale, has become quite popular as a “superfood” and appears on numerous “lists of the healthiest vegetables” in popular culture. Historically, since the last century BC, kales have been grown throughout Europe and they were mentioned in literature by both the Greeks and the Romans. At the turn of the 19th century, David G. Fairchild traveled to expand the American diet and this American botanist is credited with introducing kale to America. The vegetable was brought from the coast of the Austro-Hungarian Empire, where it was consumed as one of the principal foods of the Dalmatians [2]. Nowadays, the USA even celebrates a day dedicated to it called National Kale Day [3]. The term “kale” covers different varieties, such as: curly kale (*B. oleracea* L. convar. *acephala* var. *sabellica*), collard (*B. oleracea* L. convar. *acephala* var. *viridis*), palm kale (*B. oleracea* L. convar. *acephala* (DC.) Alef. var. *palmifolia* L.), and marrow stem kale (*B. oleracea* L. convar. *Acephala* (DC.) Alef. var. *medullosa* L.) [3,4]. Kale’s younger, more delicate leaves are used for human consumption, whilst its older leaves are better suited for use as animal fodder [5]. The most common ways to eat kale leaves are raw in salads and as kale juice, as well as cooked in a variety of soups, omelets, and stir-fries. Recently, kale sprouts have become more well-liked in the culinary world. Additionally, dried kale, also referred to as “kale chips”, has become very popular, despite the fact that drying significantly decreases the vegetable’s phytochemical and nutritious content [6]. The *acephala* variety grown traditionally in southeastern Europe (Dalmatia, Herzegovina, and Montenegro) exists in two forms: f. *viridis*, with smooth leaves, which is most often called raštika or raštan (collard greens) and f. *sabellica*, with curly leaves, which is called curly kale. These are especially popular during the winter, stewed with potatoes, root vegetables, smoked mutton (kaštradina), or cured pork meat [7]. Considering the genetic diversity between 72 different varieties of kale and cabbage, recent research making use of the single nucleotide polymorphism approach suggests that non-curled American kales should be correctly treated as collards (var. *viridis*) and not considered a kale group [4].

Sulfur-containing phytochemicals known as glucosinolates (GSLs) found in *Brassica* vegetables are primarily responsible for their health benefits. The biological activity of GSLs is attributed to their breakdown products, mostly isothiocyanates, which are obtained through the enzymatic conversion of GSLs by endogenous myrosinase (EC 3.2.1.147). Compared to other *Brassica* vegetables (such as cabbage, Brussels sprouts, broccoli, and cauliflower, for example), collard has not been the subject of as much research [8]. These studies included fresh collard leaves, in which the most prevalent GSLs were indolic ones, i.e., indol-3-ylmethyl GSL (glucobrassicin, **43**), followed by 4-hydroxyindol-3-ylmethyl GSL (4-hydroxyglucobrassicin, **28**), 4-methoxyindol-3-ylmethyl GSL (4-methoxyglucobrassicin, **48**), and *N*-methoxyindol-3-ylmethyl GSL (neoglucobrassicin, **47**), together with aliphatic GSLs (*R*_S_)-3-(methylsulfinyl)propyl GSL (glucoiberin, **73**) and allyl GSL (sinigrin, **107**) [8,9].

The majority of *Brassica* vegetables are consumed after domestic cooking, which results in myrosinase inactivation. Next to blanching and cooking, due to its convenience and effectiveness, microwave food processing has gained popularity. Unlike conventional heating methods, microwaves allow materials to immediately absorb energy and convert it to heat [10]. Because myrosinase is promptly inactivated in most procedures, heat degradation is one of the main mechanisms causing GSL loss during food processing, next to GSLs leaching into the cooking water. In addition to isothiocyanates, other breakdown products such as nitriles are thermally produced, which is undesirable in food processing, because of their potentially harmful effects [11].

The purpose of this work was to identify and quantify GSLs from different plant parts of raštika (collard, *Brassica oleracea* convar. *acephala* var. *viridis*) by their desulfo-counterparts using UHPLC-DAD-MS/MS and investigate the effect of microwaves on the formation of volatiles from the GSLs present. Furthermore, in the past decade, there has been an increased interest for the use of natural products in different fields related to cancer, either in beneficial diets or as adjuvant therapy. Current therapies in cancer treatment have numerous limitations such as high toxicity, side effects, etc. On the other hand, biomolecules from natural products, with their low level of toxicity to normal cells, show a promising potential as supplementary therapies for treating cancer. Therefore, the obtained volatile isolates from collard were subjected to MTT testing to determine their cytotoxic effects against the human cancer cells bladder cancer cell line T24 and breast cancer cell line MDA-MB-231.

## 2. Results and Discussion

**Figure 1:**
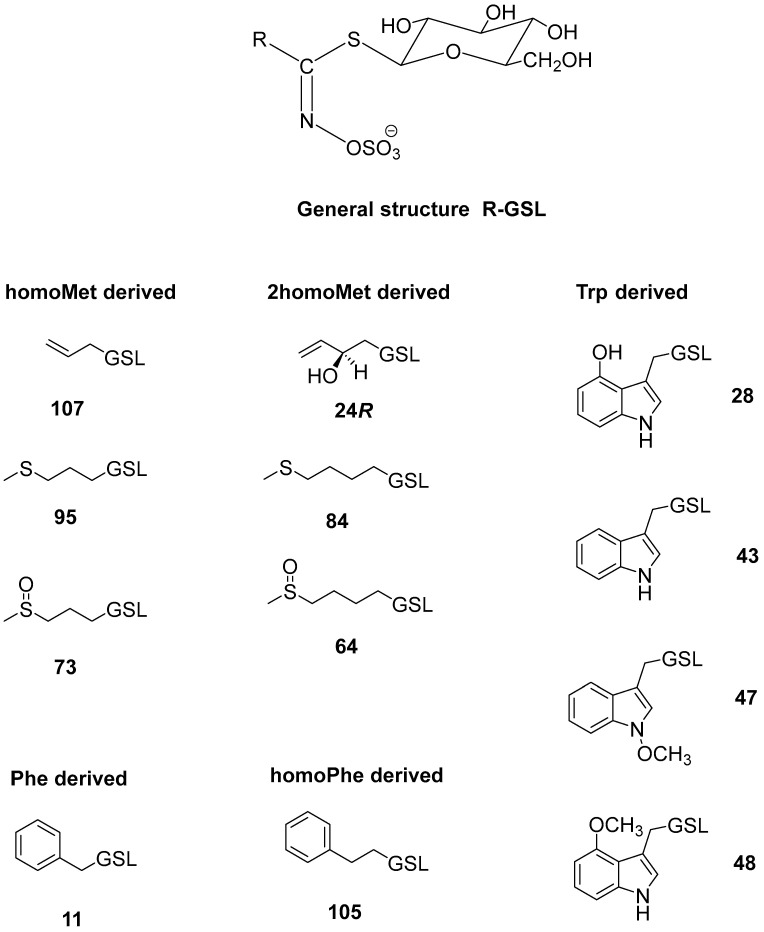
Structures of the glucosinolates (GSLs) identified in the investigated *Brassica oleracea* convar. *acephala* var. *viridis* (*cf*. Table 1): benzyl GSL (glucotropaeolin, **11**), (2*R*)-2-hydroxybut-3-enyl GSL (progoitrin, **24*R***), 4-hydroxyindol-3-ylmethyl GSL (4-hydroxyglucobrassicin, **28**), indol-3-ylmethyl GSL (glucobrassicin, **43**), *N*-methoxyindol-3-ylmethyl GSL (neoglucobrassicin, **47**), 4-methoxyindol-3-ylmethyl GSL (4-methoxyglucobrassicin, **48**), (*R*_S_)-4-(methylsulfinyl)butyl GSL (glucoraphanin, **64**), (*R*_S_)-3-(methylsulfinyl)propyl GSL (glucoiberin, **73**), 4-(methylsulfanyl)butyl GSL (glucoerucin, **84**), 3-(methylsulfanyl)propyl GSL (glucoibervirin, **95**), 2-phenylethyl GSL (gluconasturtiin, **105**), allyl GSL (sinigrin, **107**).

In this study, different plant parts of collard were investigated (Figure S1) using a microwave hydrodiffusion and gravity setup (Figure S2). Twelve GSLs were detected via UHPLC-DAD-MS/MS analyses (Figure 1 and Figure S3, and Table 1). The MS^2^ spectra are given in Figure S4.

According to the UHPLC-DAD-MS/MS analysis, aerial parts differ from the root both qualitatively and quantitatively. Aerial parts (flower, leaf and stem) contain four Met-derived GSLs and four Trp-derived GSLs ranging from 0.07 to 3.86 μmol/g DW and 0.73 to 2.57 μmol/g DW, respectively. These GSLs were previously reported in the leaves of the fresh collard investigated [8,9] as well as in other types of kale [13,14,15,16,17,18,19]. The stem was also shown to contain minor quantities of two Phe-derived GSLs. 3-(Methylsulfanyl)propyl GSL (glucoibervirin, **95**), which is formed after elongation of Met to homoMet in the core structure biosynthetic pathway, was detected only in the root. Further chain modification by flavin-monooxygenase leads to the formation of (*R*_S_)-3-(methylsulfinyl)propyl GSL (glucoiberin, **73**) (detected in all plant parts). Formation of allyl GSL (**107**), detected in the stem and root, is regulated by the AOP2 genes. Elongation of homoMet into 2homoMet results in the formation of 4-(methylsulfanyl)butyl GSL (glucoerucin, **84**, detected only in the root), and further sulfur oxidation in **84** leads to (*R*_S_)-4-(methylsulfinyl)butyl GSL (glucoraphanin, **64**, detected in the flower). But-3-enyl GSL was not previously detected in collards [8,9], which is consistent with our results, despite having been previously reported in kale leaves [13,16,17,18,19]. The critical metabolic step in *Arabidopsis* is controlled by the GS-OH gene, which has important ecological and agronomic implications [20,21,22]. This can yield from but-3-enyl GSL both the epimers of 2-hydroxybut-3-enyl GSL. The stem contains the 2*R*-epimer (progoitrin, **24*R***). As far as the authors know, the 2*S*-epimer (epiprogoitrin, **24*S***) was not detected in collard. The expression of GS-OH homologue (known as GSL-OH) is suggested in *B. oleracea* and correlated with higher levels of 2-hydroxybut-3-enyl GSL, though its biosynthesis is more stereoselective than in *A. thaliana* [22,23]. Trp-derived GSLs include 4-hydroxyglucobrassicin (**28**), glucobrassicin (**43**), 4-methoxyglucobrassicin (**48**), and neoglucobrassicin (**47**). All these GSLs were previously reported in the leaves of kales and collard. The indole ring of **43** is hydroxylated by CYP81Fs in positions 4 or 1, and IGMTs convert hydroxyl groups into methoxy groups [24,25,26,27,28,29,30].

In comparison to the aerial part, roots were shown to contain much higher levels of total GSLs (63.40 μmol/g DW) including three Met-derived and three Trp-derived GSLs, as well as two Phe-derived GSLs. Gluconasturtiin (**105**) represented the total Phe-derived GSL content (34.02 μmol/g DW) next to traces of glucotropaeolin (**11**). Generally, only GSLs from collard and kale leaves were the focus of previous reports and Phe-derived GSLs were scarcely detected and in low yield when compared to aliphatic and indole ones [13,15].

GSLs were subjected to thermal degradation using the microwave hydrodiffusion and gravity technique (MHG). Isothiocyanates and nitriles, the main volatiles originating from GSL degradation, were identified via GC-MS. (Table 2).

Generally, microwave-assisted isolation enabled degradation of GSLs, although in a low yield (2.0 to 4.3 µg/g FW). The volatiles included degradation products of three aliphatic GSLs, i.e., allyl isothiocyanate from **107**, 4-(methylsulfanyl)propyl isothiocyanate and 4-(methylsulfanyl)butanenitrile from **95**, 4-(methylsulfanyl)butyl isothiocyanate and 5-(methylsulfanyl)pentanenitrile from **84** and of one arylaliphatic GSL, and 2-phenylethyl isothiocyanate and 3-phenylpropanenitrile from **105**.

**Figure 2:**
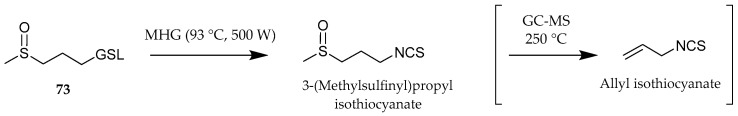
Degradation of glucoiberin (**73**) to 3-(methylsulfinyl)propyl isothiocyanate present in MHG and their thermolysis products during GC-MS analysis (not present in MHG).

Leaf volatile extract contains allyl isothiocyanate (Table 2), which can originate from sinigrin (**107**). However, the only aliphatic GSL identified using UHPLC-DAD-MS/MS was glucoiberin (**73**) (Table 1). It has previously been demonstrated that methylsulfinylalkyl isothiocyanates are not stable under severe thermal conditions during GC-MS analysis and undergo sulfenic acid elimination to afford alkenyl isothiocyanates [31]. It may therefore be concluded that allyl isothiocyanate is an artifact of 3-(methylsulfinyl)propyl isothiocyanate (Figure 2).

**Figure 3:**
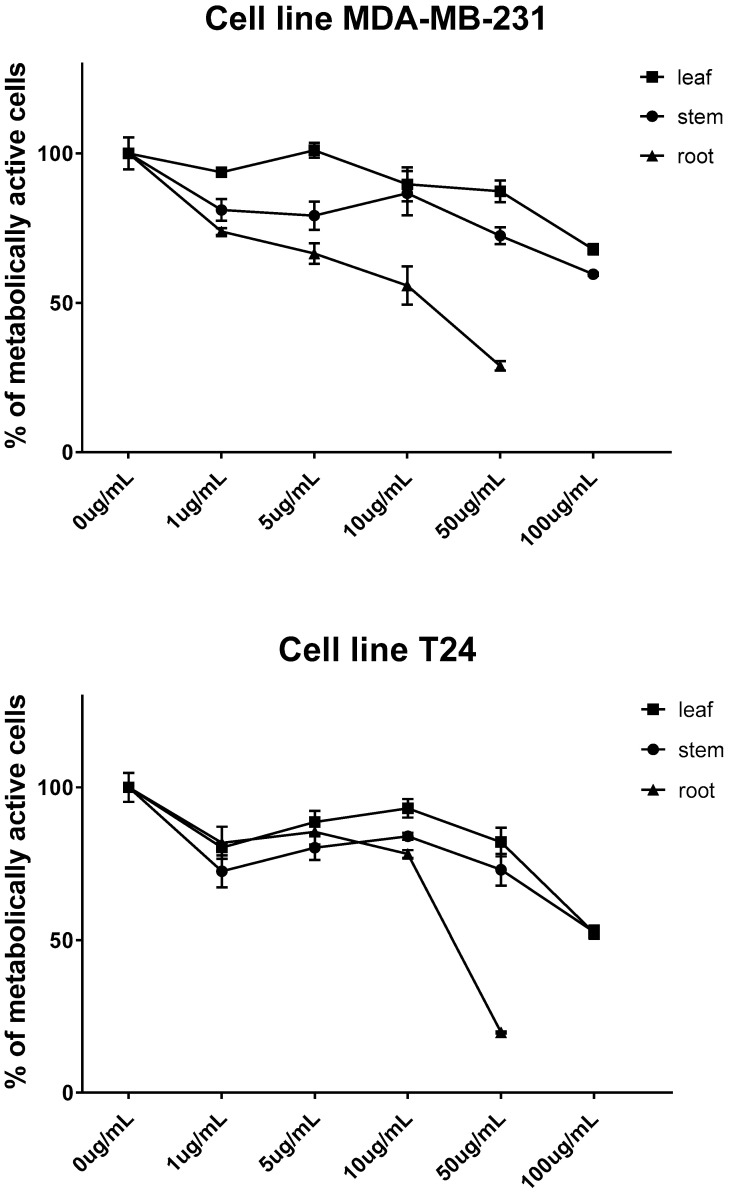
Cytotoxic effect of MHG volatiles obtained from leaf, stem, and root against T24 and MDA-MB-231. Each data point is presented as mean ± SD (*n* = 3).

The cytotoxic effect of MHG volatiles obtained from leaves, stems, and roots after 72 h of treatment was evaluated against the human bladder cancer cell line T24 and the breast cancer cell line MDA-MB-231 (results shown in Figure 3). With IC_50_ values of 21.58 µg/mL and 11.62 µg/mL against the T24 and MDA-MB-231 cell lines, respectively, root volatiles were shown to have the best cytotoxic activity, and both can be regarded as very active. In comparison to MDA-MB-231 (67.92% and 59.59% of live cells at 100 µg/mL, respectively), MHG extracts from the leaf and stem had a higher cytotoxic effect on the T24 cell line (52.33% and 52.57% of live cells at 100 µg/mL, respectively). The observed activity can generally be attributed to the breakdown products of the present GSLs, mostly isothiocyanates. Thus, the activity of the root extract with the highest content of isothiocyanates (50.72%) may be attributed to its main component, 2-phenylethyl isothiocyanate (46.18%).

The stem volatile extract activity (containing 24.53% isothiocyanates) may also be attributed to allyl isothiocyanate next to 2-phenylethyl isothiocyanate (11.57% and 12.96%, respectively). On the other hand, the leaf extract activity may be ascribed to the presence of 3-(methylsulfinyl)propyl isothiocyanate (40.16% in extract as suggested with the identified allyl isothiocyanate, Figure 2).

Volatile extracts from Brassicaceae plants containing isothiocyanates demonstrated activity against various human cancer cell lines, such as: bladder UM-UC-3 (*Lepidium latifolium*, *L. graminifolium*); bladder T24 (*Armoracia rusticana*, *Sysimbrium officinale*); glioblastoma LN229 (*L. latifolium*, *L. graminifolium*); lung A549 (*A. rusticana*, *Lunaria annua*, *S. officinale*, *Lobularia lybica*); breast MDA-MB-231 (*L. annua*, *S. officinale*); breast MCF-7 (*L. lybica*); hepatic HUH-7 (*L. lybica*); colon HCT116 (*L. lybica*, *Tropaeolum majus*), cervical HeLa; and osteosarcoma U2OS (*T. majus*) [30,31,32,33,34,35,36]. The chemical reactivity of the isothiocyanate group, which can easily bind to protein, appears to be primarily responsible for the biological activity of these compounds. Few isothiocyanates have even advanced to clinical trials as drug candidates, demonstrating their high potential against a variety of cancers (lung, breast, colon, prostate, and ovary) [32,37].

## 3. Materials and Methods

### 3.1. Materials and Reagents

Plant samples of collard (*Brassica oleracea* L. convar. *acephala* var. *viridis*, Figure S1) were cultivated in Split (43°30′26″ N, 16°31′35″ E) and collected in April 2021. The leaves were blue-green, fleshy, and rounded at the base, and they were located on thickened petioles that partially enclosed the stem. The stem was upright, reaching up to 150 cm in height, and had a well-branched spindly root. The flowers had four bright yellow oval petals that were arranged in a rectangular pattern. The specimen voucher was stored under number ZOKBOAc1. Sinigrin, DEAE-Sephadex A-25 (GE Healthcare), sulfatase (type H-1 from *Helix pomatia*), and 3-(4,5-dimethylthiazol-2-yl)-2,5-diphenyltetrazolium bromide (MTT) were purchased from Sigma-Aldrich (St. Louis, MO, USA); glucotropaeolin (**11**), progoitrin (**24R**), 4-hydroxyglucobrassicin (**28**), glucobrassicin (**43**), neoglucobrassicin (**47**), 4-methoxyglucobrassicin (**48**), glucoraphanin (**64**), glucoerucin (**84**), and gluconasturtiin (**105**) were purchased from Phytoplan Diehm & Neuberger GmbH (Heidelberg, Germany). Glucoiberin (**73**) and glucoibervirin (**95**) were identified using GSLs isolated from *Iberis umbellata* [32]. All other chemicals and reagents were of analytical grade. Human cancer cell lines (bladder T24, bladder T24, and breast MDA-MB-231, acquired from the American Type Tissue Culture Collection (ATCC, Manassas, VA, USA)), were cultured in a humidified atmosphere with 5% CO_2_ at 37 °C in Dulbecco’s modified Eagle medium (DMEM, Euro-Clone, Milano, Italy) containing 4.5 g/L glucose, 10% fetal bovine serum (FBS), and 1% antibiotics (Penicillin Streptomycin, EuroClone, Milano, Italy).

Additional purification steps are required for commercial sulfatase. Absolute ethanol was mixed with ultrapure water (30 mL) and 10 kU of aryl sulfatase (30 mL). The mixture was then centrifuged at 2650× *g* for 20 min at room temperature. The supernatant was dissolved in ethanol (90 mL). The mixture was further centrifuged at 1030× *g* for 15 min at room temperature, and the supernatants were removed and discarded. The combined pellets were thoroughly vortexed in ultrapure water (25 mL), transferred into 1 mL tubes, and frozen (−20 °C).

### 3.2. Isolation and Chemical Analysis

#### 3.2.1. Isolation of Desulfoglucosinolates

GSLs were extracted from different plant parts, as previously reported [38]. To inactivate the endogenous myrosinase, plant material was ground into a fine powder, and 100 mg were extracted for 5 min at 80 °C in 2 mL MeOH/H_2_O (70:30 *v*/*v*). Each extract was loaded onto a mini-column containing 0.5 mL of GE Healthcare’s DEAE-Sephadex A-25 anion exchange resin and conditioning it with 25 mM acetate buffer (pH 5.6). Buffer solution was added to the column after it had been washed with 70% MeOH and 1 mL of ultrapure water to create the optimal desulfation conditions. Purified sulfatase in the amount of 20 µL (0.35 U/mL) was placed into each mini-column and allowed to stand for 18 h at room temperature. The desulfoGSLs were then eluted with 1.5 mL of ultra-pure H_2_O, lyophilised and diluted to 1 mL. The samples were kept at −20 °C until UHPLC-DAD-MS/MS analysis.

#### 3.2.2. UHPLC-DAD-MS/MS Analysis

UHPLC-DAD-MS/MS (Ultimate 3000RS with TSQ Quantis MS/MS detector, Thermo Fisher Scientific, Waltham, MA, USA) using Hypersil GOLD C18 columns (3.0 µm, 3.0 × 100 mm, Thermo Fisher Scientific, Waltham, MA, USA) was used for the analysis. A gradient consisting of solvent A (50 μM NaCl in H_2_O) and solvent B (acetonitrile:H_2_O 30:70 *v*/*v*) was applied at a flow rate of 0.5 mL/min as follows: 0.14 min 96% A and 4% B; 7.84 min 14% A and 86% B; 8.96 min 14% A and 86% B; 9.52 min 5% A and 95% B; 13.16 min 5% A and 95% B; 13.44 min 96% A and 4% B; 15.68 min 96% A and 4% B. The injection volume was 5 µL, and the column temperature was maintained at 15 °C. The electrospray interface was a H-ESI source operating at 350 °C with 3.5 kV of capillary voltage, and the ion transfer tube was set at 325 °C. The system was operated in the positive ion mode with a mass range of *m/z* 150–800, scan rate of 1000 (Da/s), and resolution of 0.4 (FWHM). Nitrogen was used, and the sheath gas was set at 5.58 L/min, the aux gas at 7.97 L/min, and the sweep gas at 1.5 L/min. MS^2^ parameters included Q1 resolution 0.4 (FWHM), Q3 resolution 0.4 (FWHM), and CID Gas 1.5 (mTorr). MS^2^ analysis of each visually detected peak was performed with a systematic search for m/z values of dGSLs’ Na^+^ adducts, along with characteristic MS^2^ fragments (given in Supplementary). The signals were recorded at 227 nm using a DAD detector. Peaks of GSLs were quantified from UV peak areas using a calibration curve of pure desulfosinigrin solution with a concentration range from 0.14 to 1.4 mM (R^2^ = 0.98, y = 0.019x + 0.997) and response proportionality factors (RPFs) for each individual desulfoGSL. The following RPF values were used to quantify desulfoGSLs: 0.95 for **11**, 1.09 for **24R**, 0.28 for **28**, 0.29 for **43**, 0.20 for **47**, 0.25 for **48**, 1.07 for **64** and **73**, 1.04 for **84**, 0.80 for **95**, and 0.95 for **105** [39].

#### 3.2.3. Isolation of Volatiles

A Milestone ”ETHOS X” microwave laboratory oven (1900 W maximum) was used for microwave-assisted isolation. This was a multimode microwave reactor of 2.45 GHz in which temperature was monitored using an external infrared sensor.

Microwave hydrodiffusion and gravity (MHG, Figure S2): A typical experiment was conducted at atmospheric pressure with 313 g, 547 g, and 110 g of fresh plant material from the leaf, stem, and root, respectively, for 15 min at 500 W. The water extract was collected and extracted using CH_2_Cl_2_, dried over anhydrous Na_2_SO_4_, and concentrated using an automated sample concentrator (VLM GmbH, Bielefeld, Germany) to a volume of 1 mL. The sample was stored at −20 °C until analysis [31].

#### 3.2.4. GC-MS Analysis

The system used consisted of a gas chromatograph, model 8890 GC, equipped with an automatic liquid injector, model 7693A, and tandem mass spectrometer (MS/MS), model 7000D GC/TQ (Agilent, Inc., Santa Clara, CA, USA). A non-polar HP-5MS UI column (30 m length, 0.25 mm inner diameter, and 0.25 µm stationary phase layer thickness) was used to analyze the samples (Agilent, Inc., Santa Clara, CA, USA). The column temperature program was first set at 60 °C for 3 min, increased to 246 °C at 3 °C/min, and kept at that temperature isothermal for 25 min. Helium served as the carrier gas, and the flow rate was 1 mL/min. The volume of the injected sample was 1 µL, and the inlet temperature was 250 °C. Other parameters were: an ionization energy of 70 eV, an ion source temperature of 230 °C, and 150 °C for the quadrupole. The transfer line temperature was set at 280 °C. Duplicate analyses were performed. The individual peaks were identified via comparison of their retention indices (relative to C_8_–C_40_ n-alkanes for HP-5MS UI column) to those from the literature and/or authentic samples, as well as by comparing their mass spectra with the literature as well as Wiley 9N08 MS (Wiley, New York, NY, USA) and NIST17 (Gaithersburg, MD, USA) mass spectral databases. The percentages in Table 2 were determined as the mean value of component percentages on the HP-5MS UI column for analyses run in duplicate.

### 3.3. Cell Viability Assay (MTT Assay)

As previously described [31], the MTT spectrophotometric test was carried out using a microplate photometer, model HiPo MPP-96 (BioSan, Riga, Latvia).

The cells were treated with collard volatile isolates from different plant parts at concentrations of 1, 5, 10, 50, and 100 µg/mL in a complete medium (in triplicate) for 72 h. After treatment, the cells were incubated with 0.5 g MTT/L at 37 °C for 2 h, the medium was removed, then DMSO was added and the mixture incubated for another 10 min at 37 °C while shaking. The degree of formazan formation, an indicator of living and metabolically active cells, was measured at 570 nm. The data were calculated in relation to the untreated control (100%) from three independent measurements. The calculation of IC_50_ values was performed using GraphPad Prism software version 7.0. The criteria used to categorize the activity against the tested cell lines was based on IC_50_ values as follows: 20 μg/mL = highly active, 21–200 μg/mL = moderately active, and 201–500 μg/mL = weakly active [32].

## 4. Conclusions

Research on *Brassica* crops has primarily focused on edible parts, which are becoming more and more crucial in the fight against chronic diseases, particularly cardio-vascular diseases and several types of cancer. Collard greens (leaf), used as a traditional meal by economically deprived locals, has recently received increased attention. According to this study, the majority of GSLs located in leaves are indolic-type (97.3% of total GSLs). The other parts that are not usually consumed also contain Met-derived GSLs, while the roots show high levels of gluconasturtiin, a breakdown product of which, 2-phenylethyl isothiocyanate, seems to play a major role in observed cytotoxic activity. Generally, parts that are not consumed (flower, stem, and root) are usually thrown away or utilized in low-added-value processes. In the food industry, recycling byproducts and recovering wastes are essential approaches. *Brassica* vegetable bioactive properties may be affected by genotype, environmental and agronomical conditions during cultivation, and processing methods. In order to support a diet rich in vegetables and anticarcinogenic nutrients, these types of studies may help in a better understanding of the distribution and variability of GSL content in this neglected cruciferous vegetable.

## Figures and Tables

**Table 1:** Quantitative analysis of glucosinolates in individual plant organs of collard.

No. *	Identified Glucosinolate	*t_R_* (min)	[M+Na]^+^	Plant Tissue (μmol/g DW)			
				Flower	Leaf	Stem	Root
Met-derived							
**73**	(*R*_S_)-3-(Methylsulfinyl)propyl GSL (glucoiberin)	1.25	366	3.36 ± 0.28	0.07 ± 0.00	tr	tr
**24*R***	(2*R*)-2-Hydroxybut-3-enyl GSL (progoitrin)	1.66	332	n.d.	n.d.	0.49 ± 0.00	n.d.
**64**	(*R*_S_)-4-(Methylsulfinyl)butyl GSL (glucoraphanin)	2.32	380	0.50 ± 0.00	n.d.	n.d.	n.d.
**107**	Allyl GSL (sinigrin)	2.57	302	n.d.	n.d.	0.40 ± 0.00	7.65 ± 1.78
**95**	3-(Methylsulfanyl)propyl GSL (glucoibervirin)	5.73	350	n.d.	n.d.	n.d.	12.43 ± 1.12
**84**	4-(Methylsulfanyl)butyl GSL (glucoerucin)	6.81	364	n.d.	n.d.	n.d.	1.88 ± 0.33
Total aliphatic				3.86 ± 0.28	0.07 ± 0.00	0.89 ± 0.00	21.96 ± 3.23
Phe-derived							
**11**	Benzyl GSL (glucotropaeolin)	6.82	352	n.d.	n.d.	0.13 ± 0.00	tr
**105**	2-Phenylethyl GSL (gluconasturtiin)	8.22	366	n.d.	n.d.	0.09 ± 0.00	34.02 ± 1.23
Total arylaliphatic				n.d.	n.d.	0.22 ± 0.00	34.02 ± 1.23
Trp-derived							
**28**	4-Hydroxyindol-3-ylmethyl GSL (4-hydroxyglucobrassicin)	5.89	407	1.99 ± 0.07	2.45 ± 0.08	0.69 ± 0.04	n.d.
**43**	Indol-3-ylmethyl GSL (glucobrassicin)	7.61	391	0.42 ± 0.08	0.12 ± 0.03	0.04 ± 0.00	0.81 ± 0.03
**48**	4-Methoxyindol-3-ylmethyl GSL (4-methoxyglucobrassicin)	8.36	421	n.d.	n.d.	0.09 ± 0.00	1.93 ± 0.63
**47**	*N*-Methoxyindol-3-ylmethyl GSL (neoglucobrassicin)	9.66	421	n.d.	n.d.	0.24 ± 0.00	4.68 ± 0.44
Total indole				2.41 ± 0.15	2.57 ± 0.11	0.73 ± 0.04	7.42 ± 1.10
Total GSLs				6.27 ± 0.43	2.64 ± 0.11	1.84 ± 0.04	63.40 ± 5.56

* No.—numbering system refers to the glucosinolate numbering given in the review paper by Blažević et al. [12]. The structures are shown in Figure 1. All chromatograms are given in Figure S3, while MS^2^ spectra are given in Figure S4. [M+Na]^+^, sodium adduct of desulfoglucosinolate; *t_R_*—retention time at the UHPLC-DAD-MS/MS conditions reported here; GSL—glucosinolate; tr—traces (<0.01 μmol/g DW); n.d.—not detected; DW—dry weight of plant material. Data are expressed as the mean value ± standard error (*n* = 3).

**Table 2:** Volatiles obtained via MHG from different plant parts of collard and identified using GC-MS.

Compound	RI	Leaf	Stem	Root
Isothiocyanates				
Allyl isothiocyanate ^a^	884	40.16	11.57	tr
3-(Methylsulfanyl)propyl isothiocyanate ^a^	1308	n.d.	n.d.	3.93
4-(Methylsulfanyl)butyl isothiocyanate (erucin) ^b^	1431	n.d.	n.d.	0.61
2-Phenylethyl isothiocyanate ^a^	1464	n.d.	12.96	46.18
Nitriles				
4-(Methylsulfanyl)butanenitrile ^b^	1084	n.d.	n.d.	11.35
5-(Methylsulfanyl)pentanenitrile ^b^	1199	n.d.	n.d.	1.01
3-Phenylpropanenitrile ^a^	1241	n.d	63.48	34.31
Others				
Dimethyl trisulfide ^a^	971	n.d.	2.60	n.d.
Hex-(4*E*)-en-1-yl acetate ^b^	1007	21.10	n.d.	n.d.
Benzyl alcohol ^a^	1034	n.d.	n.d.	tr
Phenylacetaldehyde ^a^	1045	13.07	4.59	tr
Nonanal ^b^	1103	23.83	2.14	n.d.
2-Phenylethyl alcohol ^a^	1112	n.d.	n.d.	0.50
Total sum (%)		98.16	97.34	97.89
Yield (µg/g FW)		3.8	2.0	4.3
Isothiocyanates (%)		40.16	24.53	50.72
Nitriles (%)		n.d.	63.48	46.67
Others (%)		58.00	9.33	0.50

Retention indices (RI) determined on a HP-5MS capillary column; n.d.—not detected; tr—traces (<0.01%); FW—fresh weight. ^a^—Compound identified via mass spectra and RI comparison with standard; ^b^—Compound identified via retention indices and mass spectra comparison with Wiley/NIST library.

## Data Availability

Not applicable.

## Supplementary Materials

The following are available online at https://www.mdpi.com/article/10.3390/molecules28041657/s1, Figure S1. Collard (*Brassica oleracea* convar. *acephala* var. *viridis*) plant parts used (left—whole plant, right—flower, leaf, stem and root); Figure S2. Microwave hydrodiffusion and gravity (MHG) extraction setup; Figure S3. Chromatogram of desulfoglucosinolates (dGSLs) obtained from the different plant parts of collard (*Brassica oleracea* convar. *acephala* var. *viridis*, flower, leaf, stem, root, cf. Table 1): benzyl dGSL (desulfoglucotropaeolin, d**11**), (2*R*)-2-hydroxybut-3-enyl dGSL (desulfoprogoitrin, d**24*R***), 4-hydroxyindol-3-ylmethyl dGSL (desulfo-4-hydroxyglucobrassicin, d**28**), indol-3-ylmethyl dGSL (desulfoglucobrassicin, d**43**), *N*-methoxyindol-3-ylmethyl dGSL (desulfoneoglucobrassicin, d**47**), 4-methoxyindol-3-ylmethyl dGSL (desulfo-4-methoxyglucobrassicin, d**48**), (*R*_S_)-4-(methylsulfinyl)butyl dGSL (desulfoglucoraphanin, d**64**), (*R*_S_)-3-(methylsulfinyl)propyl dGSL (desulfoglucoiberin, d**73**), 4-(methylsulfanyl)butyl dGSL (desulfoglucoerucin, d**84**), 3-(methylsulfanyl)propyl dGSL (desulfoglucoibervirin, d**95**), 2-phenylethyl dGSL (desulfogluconasturtiin, d**105**), allyl dGSL (desulfosinigrin, d**107**); Figure S4. MS^2^ spectra of identified desulfoglucosinolates [40].
