# Supplementary material for: Glucosinolates and Cytotoxic Activity of Collard Volatiles Obtained Using Microwave-Assisted Extraction
Source: Molecules. 2023 Feb 9;28(4):1657. doi: 10.3390/molecules28041657 (PMC9965355; doi:10.3390/molecules28041657)
Supplement: Supplementary file 1 [file molecules-28-01657-s001.zip › molecules-2213457-supplementary.pdf]

molecules

Supplementary Materials

Glucosinolates and Cytotoxic Activity of Collard Volatiles Obtained Using Microwave-Assisted Extraction

Azra Đulović[1], Franko Burčul[2], Vedrana Čikeš Čulić[3], Patrick Rollin[4], Ivica Blažević*[1]

1 University of Split, Faculty of Chemistry and Technology, Department of Organic Chemistry, Ruđera Boškovića 35, 21000 Split, Croatia
2 Department of Analytical Chemistry, Faculty of Chemistry and Technology, University of Split, Ruđera Boškovića 35, 21000 Split, Croatia
3 School of Medicine, University of Split, Šoltanska 2, 21000 Split, Croatia
4 Université d'Orléans et CNRS, ICOA, UMR 7311, BP 6759, F-45067 Orléans, France

* Correspondence: blazevic@ktf-split.hr; Tel.:+385 21 329 434

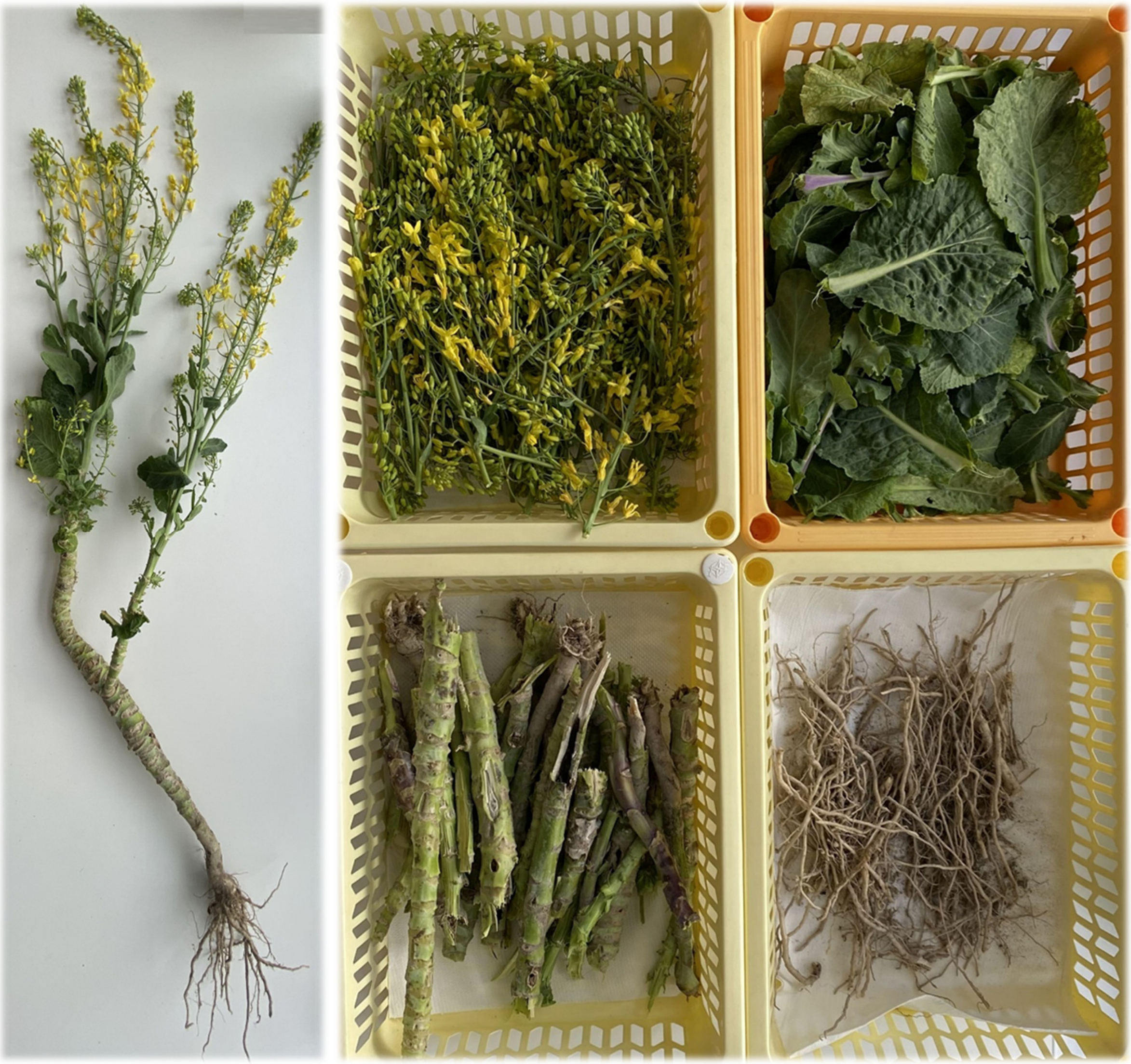

Figure S1. Collard (*Brassica oleracea* convar. *acephala* var. *viridis*) plant parts used (left - whole plant, right - flower, leaf, stem and root)

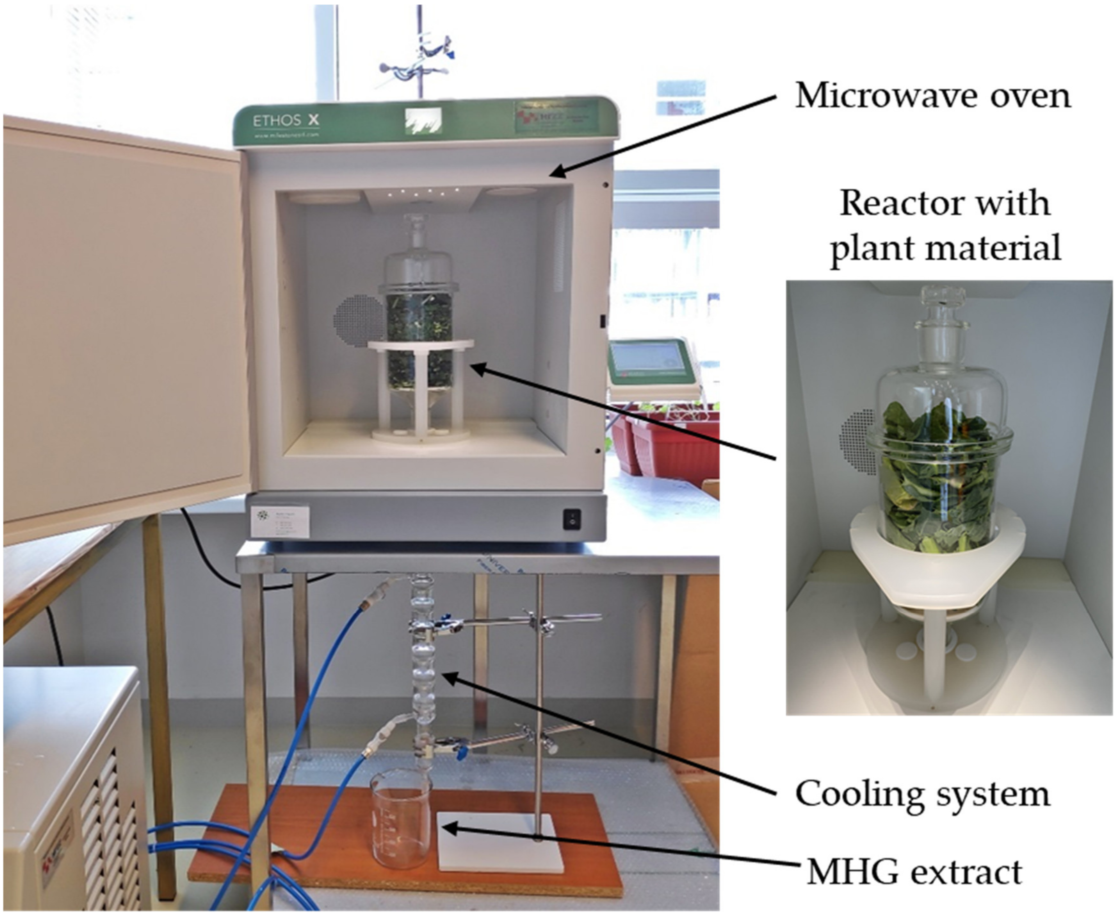

Figure S2. Microwave hydrodiffusion and gravity (MHG) extraction setup.

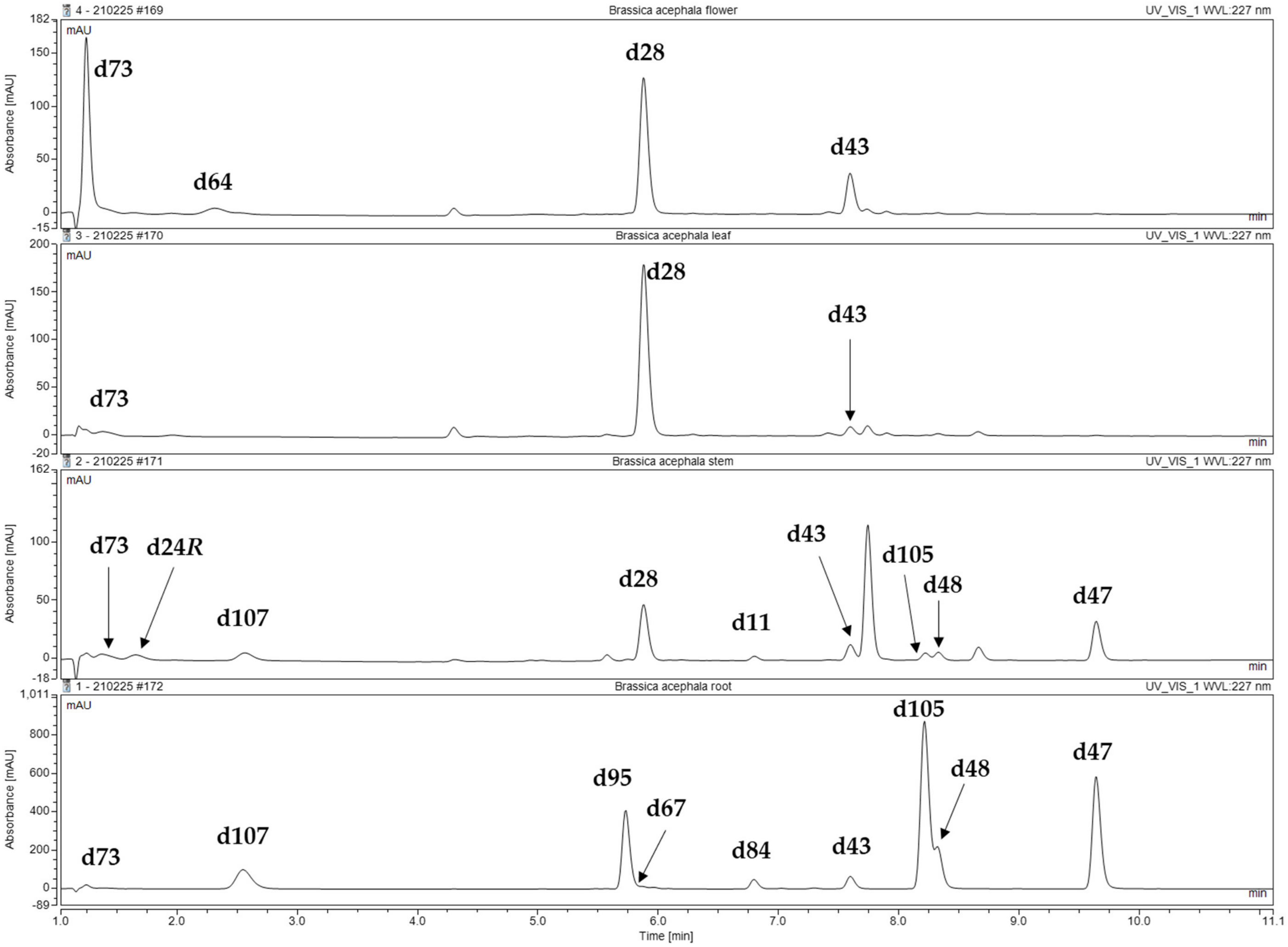

Figure S3. Chromatogram of desulfoglucosinolates (dGSLs) obtained from the different plant parts of collard (*Brassica oleracea* convar. *acephala* var. *viridis*, flower, leaf, stem, root, *cf*. Table 1): benzyl dGSL (desulfoglucotropaeolin, **d11**), (2*R*)-2-hydroxybut-3-enyl dGSL (desulfoprogoitrin, **d24*R***), 4-hydroxyindol-3-ylmethyl dGSL (desulfo-4-hydroxyglucobrassicin, **d28**), indol-3-ylmethyl dGSL (desulfoglucobrassicin, **d43**), *N*-methoxyindol-3-ylmethyl dGSL (desulfoneoglucobrassicin, **d47**), 4-methoxyindol-3-ylmethyl dGSL (desulfo-4-methoxyglucobrassicin, **d48**), (*R*s)-4-(methylsulfinyl)butyl dGSL (desulfoglucoraphanin, **d64**), (*R*s)-3-(methylsulfinyl)propyl dGSL (desulfoglucoiberin, **d73**), 4-(methylsulfanyl)butyl dGSL (desulfoglucoerucin, **d84**), 3-(methylsulfanyl)propyl dGSL (desulfoglucoibervirin, **d95**), 2-phenylethyl dGSL (desulfogluconasturtiin, **d105**), allyl dGSL (desulfosinigrin, **d107**)

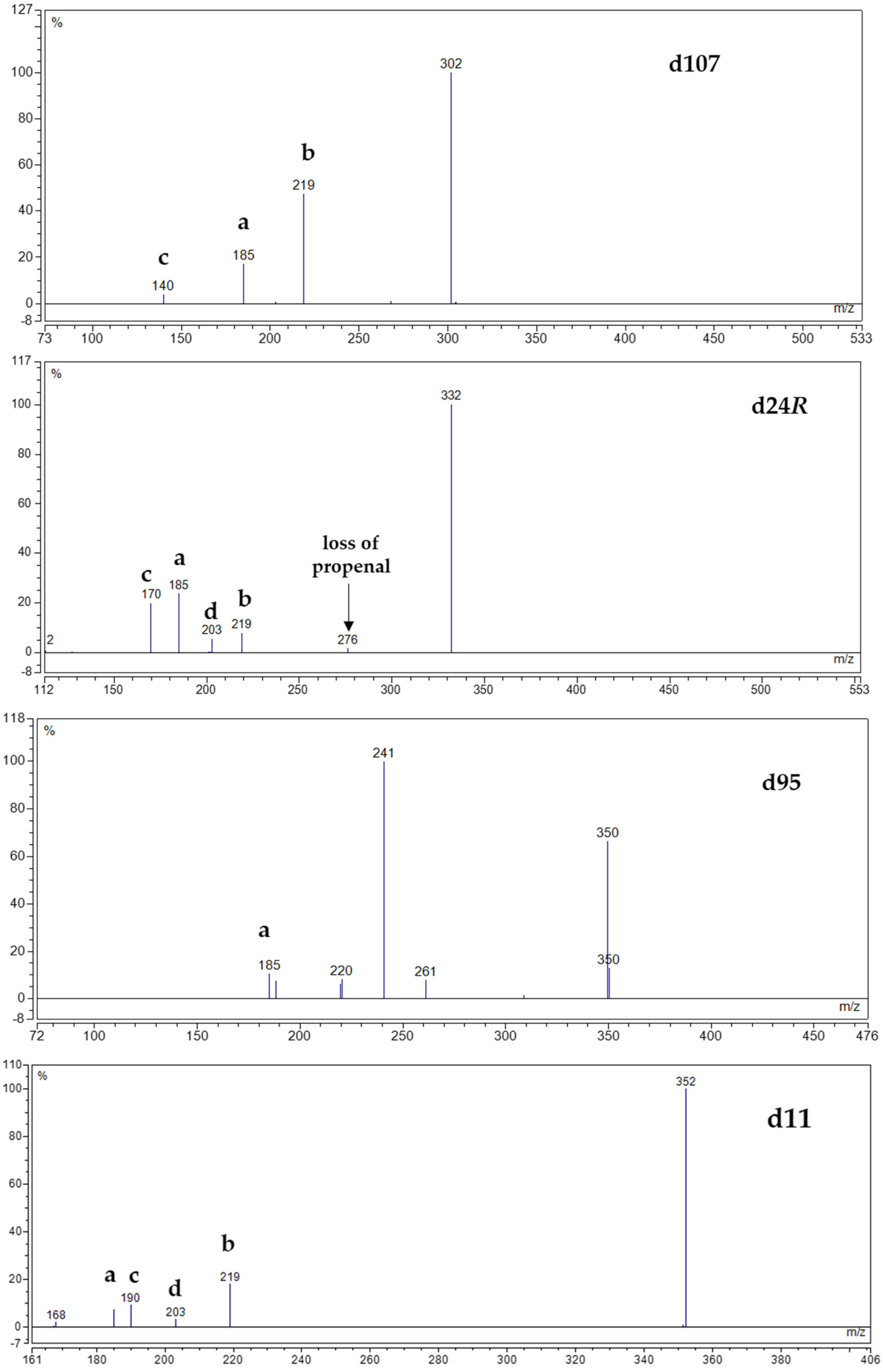

Figure S4. *Continuous*

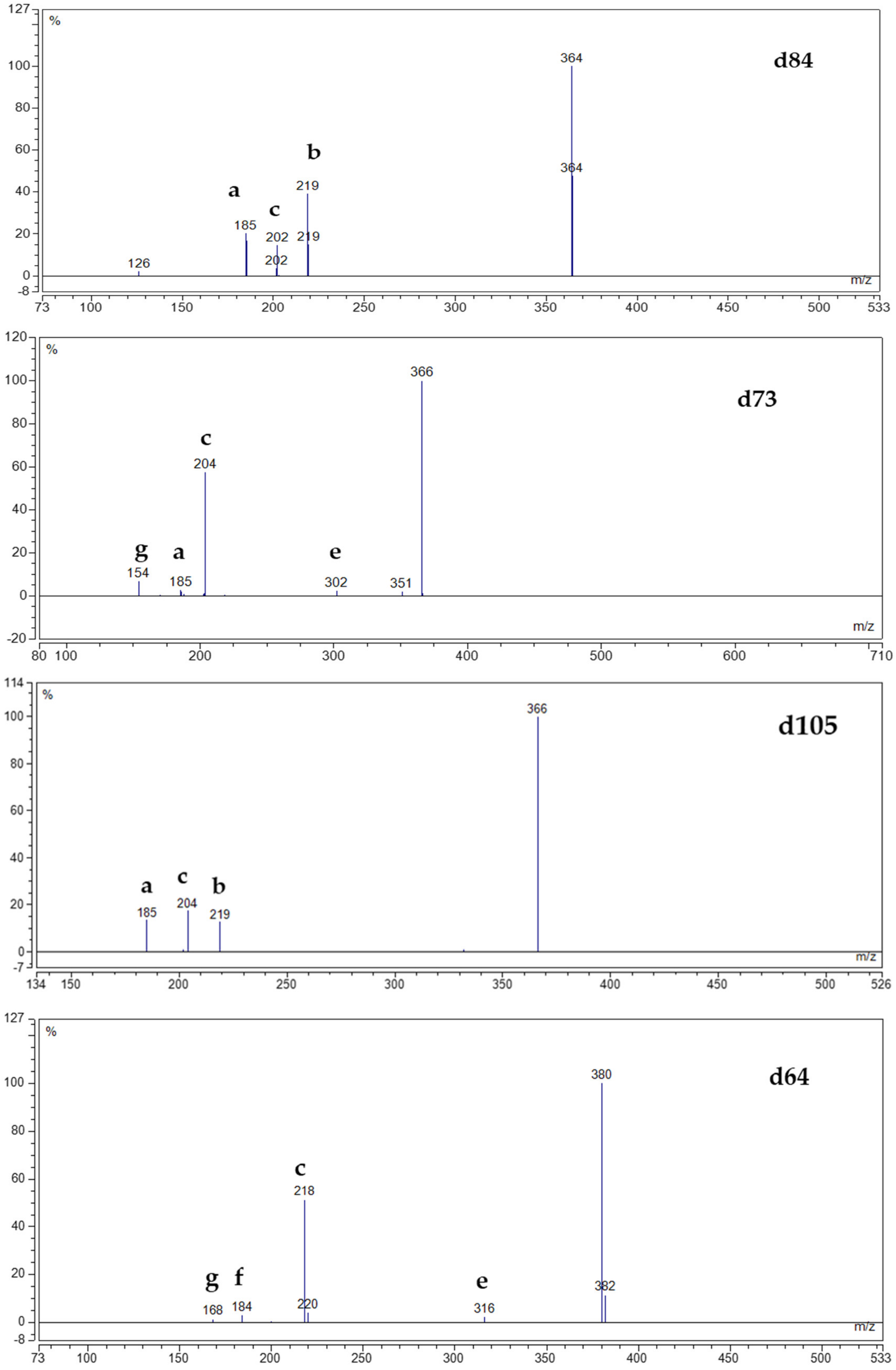

Figure S4. *Continuous*

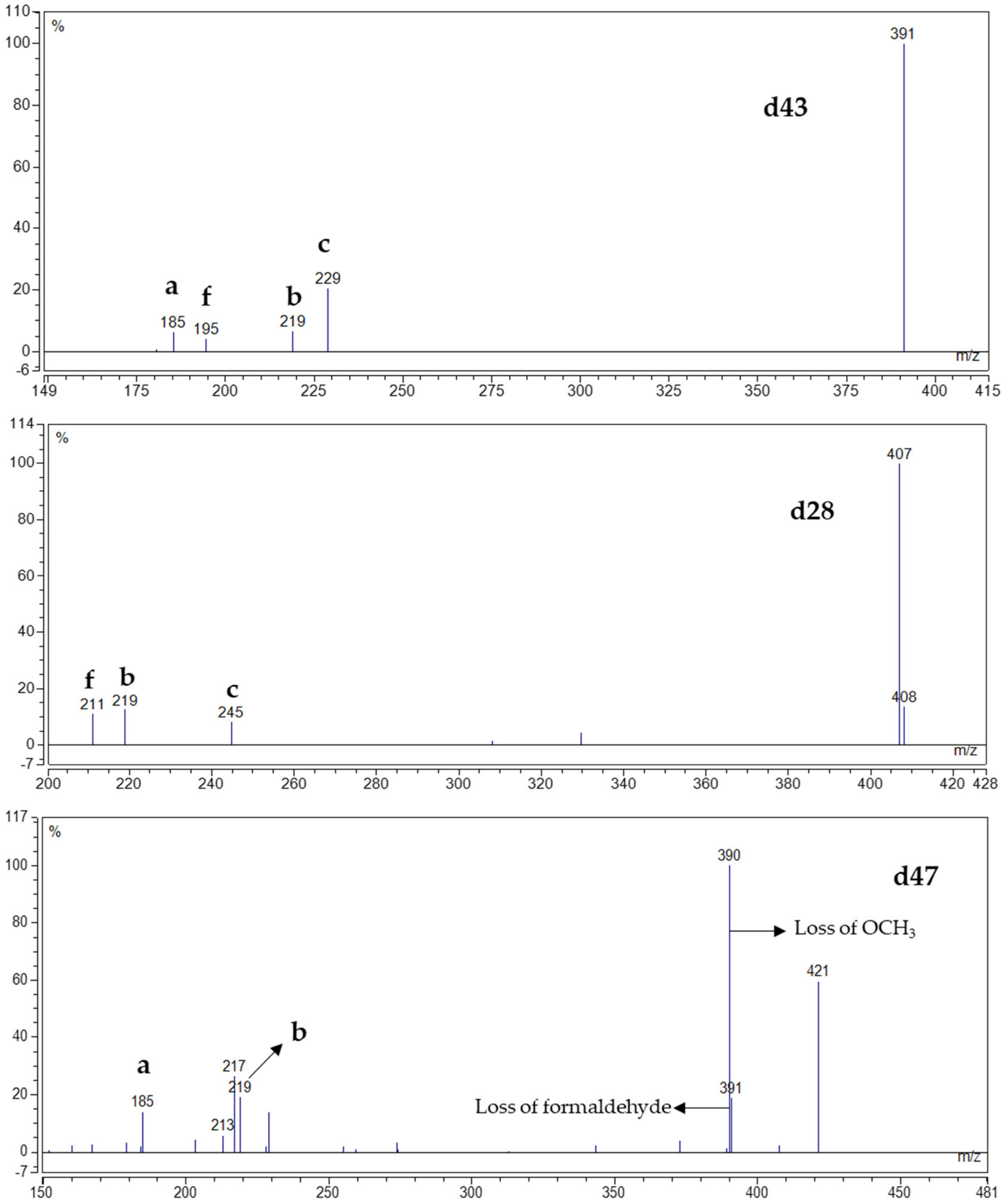

Figure S4. *Continuous*

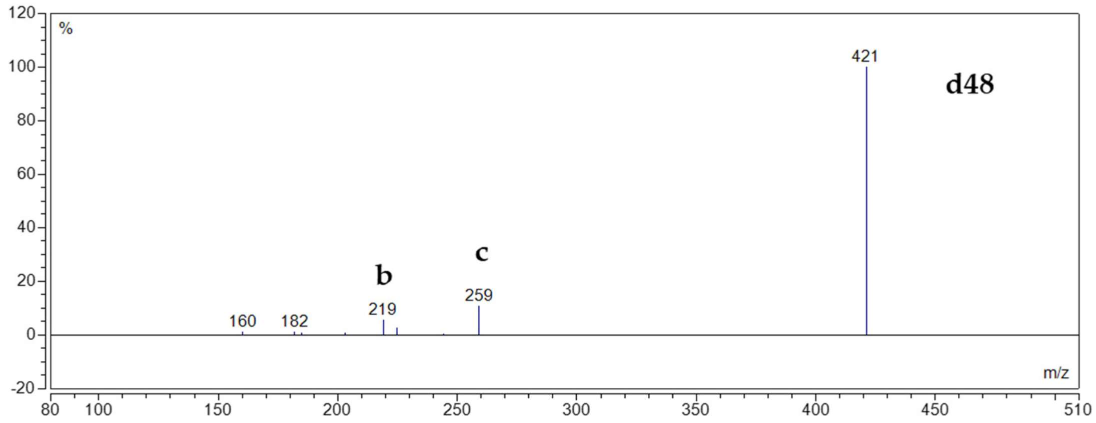

Figure S4. MS^2 spectra of identified desulfoglucosinolates. Fragments: **a** - Na^+ adduct of anhydroglucose, **b** - Na^+ adduct of thioglucose, **c** - Loss of anhydroglucose (*m*/*z* 162), **d** - Na^+ adduct of glucose, **e** - Loss of H(SO)CH_3 (*m*/*z* 64), **f** - Loss of thioglucose (*m*/*z* 196), **g** - Loss of oxidized thioglucose (*m*/*z* 212) in methylsulfinylalkyl desulfoglucosinolates **d64** and **d73** [1].
